# Health insurance among survivors of childhood cancer following Affordable Care Act implementation

**DOI:** 10.1093/jnci/djae111

**Published:** 2024-05-13

**Authors:** Anne C Kirchhoff, Austin R Waters, Qi Liu, Xu Ji, Yutaka Yasui, K Robin Yabroff, Rena M Conti, I -Chan Huang, Tara Henderson, Wendy M Leisenring, Gregory T Armstrong, Paul C Nathan, Elyse R Park

**Affiliations:** Department of Pediatrics, University of Utah, Salt Lake City, UT, USA; Cancer Control and Population Sciences Research Program, Huntsman Cancer Institute, Salt Lake City, UT, USA; Cancer Control and Population Sciences Research Program, Huntsman Cancer Institute, Salt Lake City, UT, USA; Department of Health Policy and Management, University of North Carolina, Chapel Hill, NC, USA; Department of Public Health Sciences, University of Alberta, Edmonton, AB, Canada; Department of Pediatrics, Emory University School of Medicine/AFLAC Cancer & Blood Disorders Center, Children’s Healthcare of Atlanta, Atlanta, GA, USA; Department of Epidemiology and Cancer Control, St Jude Children’s Research Hospital, Memphis, TN, USA; Department of Surveillance and Health Equity Science, American Cancer Society, Atlanta, GA, USA; Department of Markets, Public Policy, and Law, Boston University Questrom School of Business, Boston, MA, USA; Department of Epidemiology and Cancer Control, St Jude Children’s Research Hospital, Memphis, TN, USA; Department of Pediatrics, University of Chicago, Comer Children’s Hospital, Chicago, IL, USA; Clinical Research and Public Health Science Divisions, Fred Hutchinson Cancer Center, Seattle, WA, USA; Department of Epidemiology and Cancer Control, St Jude Children’s Research Hospital, Memphis, TN, USA; Department of Pediatrics and Health Policy, Division of Hematology/Oncology, The Hospital for Sick Children, The University of Toronto, Toronto, ON, Canada; Department of Psychiatry, Massachusetts General Hospital, Boston, MA, USA

## Abstract

**Background:**

The Affordable Care Act (ACA) increased private nonemployer health insurance options, expanded Medicaid eligibility, and provided preexisting health condition protections. We evaluated insurance coverage among long-term adult survivors of childhood cancer pre- and post-ACA implementation.

**Methods:**

Using the multicenter Childhood Cancer Survivor Study, we included participants from 2 cross-sectional surveys: pre-ACA (2007-2009; survivors: n = 7505; siblings: n = 2175) and post-ACA (2017-2019; survivors: n = 4030; siblings: n = 987). A subset completed both surveys (1840 survivors; 646 siblings). Multivariable regression models compared post-ACA insurance coverage and type (private, public, uninsured) between survivors and siblings and identified associated demographic and clinical factors. Multinomial models compared gaining and losing insurance vs staying the same among survivors and siblings who participated in both surveys.

**Results:**

The proportion with insurance was higher post-ACA (survivors pre-ACA 89.1% to post-ACA 92.0% [+2.9%]; siblings pre-ACA 90.9% to post-ACA 95.3% [+4.4%]). Post-ACA insurance increase in coverage was higher among those aged 18-25 years (survivors: +15.8% vs +2.3% or less ages 26 years and older; siblings +17.8% vs +4.2% or less ages 26 years and older). Survivors were more likely to have public insurance than siblings post-ACA (18.4% vs 6.9%; odds ratio [OR] = 1.7, 95% confidence interval [CI] = 1.1 to 2.6). Survivors with severe chronic conditions (OR = 4.7, 95% CI = 3.0 to 7.3) and those living in Medicaid expansion states (OR = 2.4, 95% CI = 1.7 to 3.4) had increased odds of public insurance coverage post-ACA. Among the subset completing both surveys, low- and mid-income survivors (<$40 000 and <$60 000, respectively) experienced insurance losses and gains in reference to highest household income survivors (≥$100 000), relative to odds of keeping the same insurance status.

**Conclusions:**

Post-ACA, more childhood cancer survivors and siblings had health insurance, although disparities remain in coverage.

Due to ongoing improvements in primary cancer therapy and supportive care, most children with cancer survive into adulthood as more than 85% become 5-year survivors (1). Today, there are more than 500 000 survivors of childhood cancer living in the United States (2). However, childhood cancer survivors are at risk for multiple morbidities and premature mortality from their cancer and its therapy in the decades following diagnosis as 96% develop a severe or life-threatening health condition and their excess mortality risk persists for 40 or more years from diagnosis (3,4). Thus, adequate health insurance coverage is fundamental to survivors’ ability to access appropriate follow-up care.

The Patient Protection and Affordable Care Act (ACA) was passed in 2010 with the intention of increasing access to affordable, quality health insurance and with specific options to protect individuals with preexisting conditions, such as survivors of childhood cancer (5-7). The ACA extended employer-sponsored parents’ private coverage for dependents up to age 26 years starting in 2010. Most ACA provisions were implemented in 2014, including the health insurance marketplace with subsidized coverage and premium tax credits for insurance purchase and expanded Medicaid income eligibility (6,7). Prior to the ACA, childhood cancer survivors were more likely to be uninsured and underinsured than their siblings or individuals without a cancer history (8-10).

Although uninsurance has declined since ACA implementation, little is known about whether the quality of insurance coverage for adult survivors of childhood cancer has improved (11,12). Today, comprehensive health insurance remains unaffordable for many middle- to low-income cancer survivors in the United States because of limits in insurance subsidies and because some states have not expanded Medicaid coverage to residents (9). Even among individuals who have insurance coverage, coverage disruptions and underinsurance (having continuous coverage but still experiencing high out-of-pocket costs relative to income, which with the increase in high deductible plans may lead to greater cost pressures for survivors) remain common in the general population (13,14). Whether the ACA has improved perceptions of insurance stability, which could affect survivors’ health-care-seeking behaviors regardless of actual insurance interruptions, as well as underinsurance, is unknown (15).

Drawing from the nationwide Childhood Cancer Survivor Study (CCSS), this analysis had the objective to investigate health insurance coverage for childhood cancer survivors compared with a similar age and demographic sample of siblings, approximately 10 years into implementation of the ACA. We hypothesized that survivors and siblings would have higher levels of coverage but that survivors would be more likely to have public insurance coverage than siblings after the ACA implementation. In addition, we examined underinsurance and worry about insurance stability among survivors and siblings. We also sought to identify whether among survivors only, certain subgroups of survivors such as those with lower incomes and with chronic health conditions remain uninsured or underinsured after the ACA implementation.

## Methods

### Population

The CCSS design has been reported in detail (16,17). The CCSS is a retrospective cohort study with longitudinal follow-up of survivors of childhood cancer originally diagnosed between 1970 and 1999 before the age of 21 years who survived at least 5 years after their original diagnosis and a randomly selected sibling sample to provide a comparison sample similar in age and demographics. The original CCSS cohort (diagnosed 1970-1986) and expansion cohort (1987-1999) are evaluated as a single, harmonized cohort that receives survey-based follow-up every 2-3 years. In the expansion cohort, acute lymphoblastic leukemia survivors were undersampled because of its dominant size. Institutional review boards at each of 31 participating sites in the United States and Canada approved the study, and informed consent was obtained from participants. Analyses were limited to US residents. Reporting follows the Strengthening the Reporting of Observational Studies in Epidemiology guidelines (18).

### Exposures and outcome measures

We evaluated responses from 2 cross-sectional CCSS surveys that assessed health insurance. The first was administered pre-ACA (2007-2009) and was limited to the original cohort. The second was administered post-ACA (2017-2019) and included original and expansion cohorts (19). Because of the sensitive nature of the post-ACA survey with questions about financial toxicity (19), a randomly selected, representative sample of one-third of eligible participants received the questions about insurance coverage.

First, we examined insurance coverage pre- and post-ACA by comparing 2 cross-sectional populations using the 2007-2009 survey (survivors: n = 7505; siblings: n = 2175) and the 2017-2019 survey participants (survivors: n = 4030; siblings: n = 987). Any insurance coverage was collected at both timepoints, whereas type of insurance (private, public) was only ascertained in 2017-2019.

Second, among participants who completed the 2017-2019 survey, we examined type of insurance coverage (public, private, uninsured) and, among insured participants, both underinsurance and perceived insurance stability. Underinsurance was defined as spending more than 10% of household income on out-of-pocket medical expenses (20). Perceived insurance stability was generated from an item that asked about participants’ concern regarding maintaining current level of insurance coverage, grouped as not concerned, a little concerned, vs moderately greatly concerned (21).

Third, to investigate insurance loss and gains, we conducted a longitudinal analysis limited to the original cohort of survivors (n = 1840) and siblings (n = 646) who were randomly selected and participated in both the pre- and post-ACA surveys. Insurance loss or gain was operationalized as no change in insurance, losing insurance, and gaining insurance pre- and post-ACA.

Other measures included demographic factors and chronic conditions using the National Cancer Institute’s Common Terminology Criteria for Adverse Events (scored as 1 = mild; 2 = moderate; 3 = severe; and 4 = life-threatening, grouped as none vs 1-2 and 3-4) (22) from CCSS surveys. Cancer treatment was abstracted from medical records of survivors who authorized release. Residence in Medicaid expansion state was ascertained based on residence as of the post-ACA survey.

### Statistical approach

We tabulated demographic characteristics of survivors and siblings and diagnostic and treatment characteristics of survivors for the pre- and post-ACA samples. For the cross-sectional analyses that compared insurance coverage pre- and post-ACA, we examined the unadjusted proportion of insured participants and the change between the 2 timepoints among subgroups (age at survey, sex, race and ethnicity) for survivors and siblings, respectively. Multivariable logistic regression examined pre-post changes in any insurance coverage for the 2 cross-sectional samples, with an interaction between survivor and sibling and pre- and postsurvey included to allow examination of the pre-post change differences between survivors and siblings, adjusting for age at survey, sex, and race and ethnicity.

For the post-ACA analyses, type of insurance (public, private, and uninsured) was examined in a multinomial logistic regression comparing survivors and siblings, estimating adjusted odds ratios (ORs) of privately insured (vs uninsured) and publicly insured (vs uninsured), adjusting for age at survey, sex, and race and ethnicity. Two multivariable logistic regression models evaluated underinsurance and perceived insurance stability between survivors and siblings who were currently insured, adjusting for age at survey, sex, and race and ethnicity.

We then fit a multinomial logistic regression model limited to survivors to assess associations of insurance type with demographic and clinical (treatment-related risk factors) characteristics with uninsured as the reference. Similarly, multivariable logistic regression models evaluated demographic and clinical characteristics among survivors associated with underinsurance and perceived insurance stability as based on previous literature (19,21,23). All models that evaluated demographic and treatment risk factors adjusted for residence in a state that had implemented Medicaid expansion or not by the survey date. Variables with a *P* value less than .2 in univariate models were then included in the multivariable models. For these models, because the ACA-dependent coverage option until age 26 years, we examined differences by age at survey, examining those aged 19-25 years compared with older age groups.

The longitudinal analysis used multinomial logistic regression models to evaluate associations of demographic and clinical characteristics with insurance losses or gains (reference group: no change) for survivors and siblings, respectively, with variables with a *P* value less than .2 in univariate models. We also examined pre-post changes in any insurance coverage for the longitudinal samples, with an interaction between survivor and sibling and pre and post survey, adjusting for age at survey, sex, and race and ethnicity. For these models, chronic conditions and household income are from the pre-ACA survey; age and Medicaid expansion status are based on the post-ACA survey (using residential zip code at survey); race and ethnicity, age at diagnosis, and treatment information come from the baseline survey and/or medical records. Further, the household income category pre-ACA and post-ACA were used to create a change in household income variable (ie, no change, decrease, increase). As a supplemental analysis, change in income was then used instead of household income in multinomial logistic regression models to evaluate associations of change in income as well as demographic and clinic characteristics with insurance losses or gains.

Generalized estimating equations were used in the above regression analyses to account for potential within-family correlation and correlation between the 2 timepoints of the same participants. All analyses included sampling weights for the expansion cohort acute lymphoblastic leukemia sampling. For income and race and ethnicity, we created “missing” and “unknown” categories, respectively. Because of sample size limitations, the race and ethnicity variables were collapsed into an aggregate variable for the adjusted multivariable regression analyses but are reported as separate variables for the unadjusted comparisons. Analyses using treatment were restricted to survivors whose treatment information was available. Statistical significance was preset at a *P* value less than .05. All statistical tests were 2-sided.

## Results

### Demographic and treatment characteristics

The pre-ACA survey included 7505 survivors and 2175 siblings, and the post-ACA survey included 4030 survivors and 987 siblings. A total of 1840 survivors and 646 siblings completed both surveys. Participants were approximately half women (pre-ACA survivor = 50.2%, sibling = 53.7%; post-ACA survivor = 51.3%, sibling = 57.6%) and primarily non-Hispanic White at both surveys (pre-ACA survivor = 86.5%, sibling = 89.5%; post-ACA survivor = 83.2%, sibling = 89.0%) and in the pre-post longitudinal sample (Table 1). Leukemia and Hodgkin lymphoma accounted for the largest proportion of cancers. Supplementary Figure 1 and Supplementary Table 1 (available online) show response rates and demographic and clinical differences, respectively, between respondents and non-respondents (participants tend to be more female and non-Hispanic White).

**Table 1.** Demographic characteristics of survivors of childhood cancer and siblings pre- (2007-2009) vs post- (2017-2019) Affordable Care Act (ACA)

Demographic and clinical characteristics	Survivors			Siblings		
	Pre-ACA survey, No. (%)	Post-ACA survey, No. (%)	Pre- and post- ACA survey,^a^ No. (%)	Pre-ACA survey, No. (%)	Post-ACA survey, No. (%)	Pre- and post-ACA survey,^a^ No. (%)
	(n = 7505)	(n = 4030)	(n = 1840)	(n = 2175)	(n = 987)	(n = 646)
Age at survey, y^b^						
18-25	572 (7.6)	309 (10.3)	0 (0)	197 (9.1)	33 (3.3)	51 (7.9)
26-29	1200 (16.0)	365 (11.4)	0 (0)	263 (12.1)	27 (2.7)	64 (9.9)
30-34	1531 (20.4)	620 (17.8)	94 (5.1)	357 (16.4)	93 (9.4)	97 (15.0)
35-39	1707 (22.7)	832 (19.4)	355 (19.3)	443 (20.4)	143 (14.5)	131 (20.3)
40-44	1353 (18.0)	692 (15.1)	393 (21.4)	395 (18.2)	168 (17)	126 (19.5)
45-49	814 (10.8)	561 (12.1)	412 (22.4)	306 (14.1)	167 (16.9)	102 (15.8)
50 and older	328 (4.4)	651 (14)	586 (31.8)	214 (9.8)	356 (36.1)	75 (11.6)
Sex						
Male	3736 (49.8)	1952 (48.7)	867 (47.1)	1008 (46.3)	418 (42.4)	274 (42.4)
Female	3769 (50.2)	2078 (51.3)	973 (52.9)	1167 (53.7)	569 (57.6)	372 (57.6)
Race and ethnicity						
Black, non-Hispanic	227 (3.0)	188 (4.9)	44 (2.4)	51 (2.3)	15 (1.5)	8 (1.2)
Hispanic	323 (4.3)	267 (7.0)	75 (4.1)	61 (2.8)	33 (3.3)	18 (2.8)
Other^c^	461 (6.1)	198 (4.9)	105 (5.7)	117 (5.4)	61 (6.2)	36 (5.6)
White, non-Hispanic	6494 (86.5)	3377 (83.2)	1616 (87.8)	1946 (89.5)	878 (89.0)	584 (90.4)
Employment status						
Working for pay	5770 (78.5)	3138 (79.5)	1470 (81.6)	1802 (84.1)	840 (85.4)	535 (83.5)
Not employed, in labor force	1577 (21.5)	862 (20.5)	332 (18.4)	340 (15.9)	144 (14.6)	106 (16.5)
Medicaid expansion state resident (2017-2019 survey)						
Yes	—	3131 (78.6)	1433 (78.5)	—	769 (80.4)	517 (80.3)
No	—	826 (21.4)	392 (21.5)	—	188 (19.6)	127 (19.7)
Household income^d^						
<$20 000	783 (10.5)	350 (9.2)	143 (7.8)	110 (5.1)	32 (3.2)	21 (3.3)
$20 000-$39 999	1257 (16.8)	457 (11.9)	305 (16.6)	265 (12.2)	67 (6.8)	63 (9.8)
$40 000-$59 999	1242 (16.6)	475 (11.4)	311 (16.9)	337 (15.5)	99 (10)	96 (14.9)
$60 000-$79 999	1114 (14.9)	451 (11.0)	290 (15.8)	341 (15.7)	93 (9.4)	103 (15.9)
$80 000-$99 999	787 (10.5)	421 (10.3)	208 (11.3)	280 (12.9)	123 (12.5)	92 (14.2)
≥$100 000	1590 (21.2)	1176 (28.2)	422 (23.0)	704 (32.4)	465 (47.1)	242 (37.5)
Missing	713 (9.5)	700 (18)	157 (8.6)	136 (6.3)	108 (10.9)	29 (4.5)
Educational status^e^						
High school or less	1197 (16)	—	255 (13.9)	244 (11.2)	—	52 (8)
Some postgraduate, college	2333 (31.1)	—	516 (28.1)	647 (29.7)	—	162 (25.1)
College graduate or more	3966 (52.9)	—	1065 (58)	1284 (59)	—	432 (66.9)
Marital status^e^						
Married or living as married	4246 (56.6)	—	1126 (61.2)	1510 (69.5)	—	478 (74.0)
Single, never Married	2525 (33.7)	—	572 (31.1)	436 (20.1)	—	113 (17.5)
Divorced, separated	708 (9.4)	—	140 (7.6)	220 (10.1)	—	53 (8.2)
Widowed	18 (0.2)	—	1 (0.1)	8 (0.4)	—	2 (0.3)
Chronic health conditions						
None	1077 (14.4)	636 (17.9)	261 (14.2)	732 (33.7)	302 (30.6)	192 (29.7)
Grade 1-2	3081 (41.1)	1689 (42.6)	814 (44.2)	1136 (52.2)	536 (54.3)	360 (55.7)
Grade 3-4	3347 (44.6)	1705 (39.5)	765 (41.6)	307 (14.1)	149 (15.1)	94 (14.6)
Age at diagnosis, y						
0-4	2993 (39.9)	1562 (41.7)	741 (40.3)	—	—	—
5-9	1630 (21.7)	904 (23.5)	399 (21.7)	—	—	—
10-14	1528 (20.4)	907 (20.5)	384 (20.9)	—	—	—
15 and older	1354 (18)	657 (14.3)	316 (17.2)	—	—	—
Diagnosis						
Leukemia	2592 (34.5)	1237 (40.1)	656 (35.7)	—	—	—
Central nervous system	881 (11.7)	672 (14.4)	186 (10.1)	—	—	—
Hodgkin lymphoma	997 (13.3)	480 (10.3)	210 (11.4)	—	—	—
Non-Hodgkin lymphoma	584 (7.8)	349 (7.5)	155 (8.4)	—	—	—
Kidney (Wilms)	684 (9.1)	386 (8.3)	187 (10.2)	—	—	—
Neuroblastoma	493 (6.6)	292 (6.3)	125 (6.8)	—	—	—
Soft tissue sarcoma	658 (8.8)	267 (5.7)	159 (8.6)	—	—	—
Bone cancer	616 (8.2)	347 (7.4)	162 (8.8)	—	—	—
Any radiation						
Yes	4547 (65.2)	2086 (50.9)	1101 (64.3)	—	—	—
No	2432 (34.8)	1724 (49.1)	612 (35.7)	—	—	—
Chest radiation						
Yes	1758 (25.7)	888 (21.4)	408 (24.3)	—	—	—
No	5076 (74.3)	2833 (78.6)	1271 (75.7)	—	—	—
Abdominal or pelvic radiation						
Yes	1804 (26.4)	852 (20.6)	418 (24.9)	—	—	—
No	5031 (73.6)	2870 (79.4)	1261 (75.1)	—	—	—
Limb (arm or leg) radiation						
Yes	287 (4.2)	187 (4.7)	75 (4.5)	—	—	—
No	6547 (95.8)	3534 (95.3)	1604 (95.5)	—	—	—
Total body irradiation						
Yes	90 (1.3)	97 (2.6)	22 (1.3)	—	—	—
No	6743 (98.7)	3624 (97.4)	1657 (98.7)	—	—	—
Any surgery						
Yes	5348 (77.4)	2982 (70.5)	1315 (77.4)	—	—	—
No	1563 (22.6)	818 (29.5)	385 (22.6)	—	—	—
Any chemotherapy						
Yes	5517 (79.7)	3157 (85.4)	1407 (82.6)	—	—	—
No	1406 (20.3)	645 (14.6)	296 (17.4)	—	—	—
Received alkylating agents						
Yes	3476 (50.4)	2056 (55.3)	865 (51.1)	—	—	—
No	3414 (49.6)	1727 (44.7)	828 (48.9)	—	—	—
Anthracycline cumulative dose						
None	4244 (63.4)	1905 (46.9)	1012 (61.6)	—	—	—
1-299 mg/m²	1549 (23.2)	1295 (41.3)	403 (24.5)	—	—	—
≥300 mg/m²	896 (13.4)	481 (11.8)	228 (13.9)	—	—	—

a Participants who completed both the 2007-2009 and 2017-2019 surveys.

b Pre-ACA, maximum age in years was 58.9 for survivors and 62.6 for siblings. Post-ACA, maximum age for survivors 67.4 and siblings 69.2. The Pre/Post-ACA sample maximum age was 57.5 for survivors and 59.5 for siblings.

c Other designates participants indicating other race groups or multiple race groups.

d For pre-ACA columns, income from 2007-2009 survey was used and for post-ACA column income from 2017-2019 survey was used. For the longitudinal sample who had surveys in both the 2007-2009 and 2017-2019 time-periods, the values at 2007-2009 are shown.

e Education and marital status were asked only in 2007-2009. For the longitudinal sample, the values at 2007-2009 are shown.

### Survivor vs sibling comparisons in health insurance coverage pre-ACA to post-ACA

**Figure 1.**
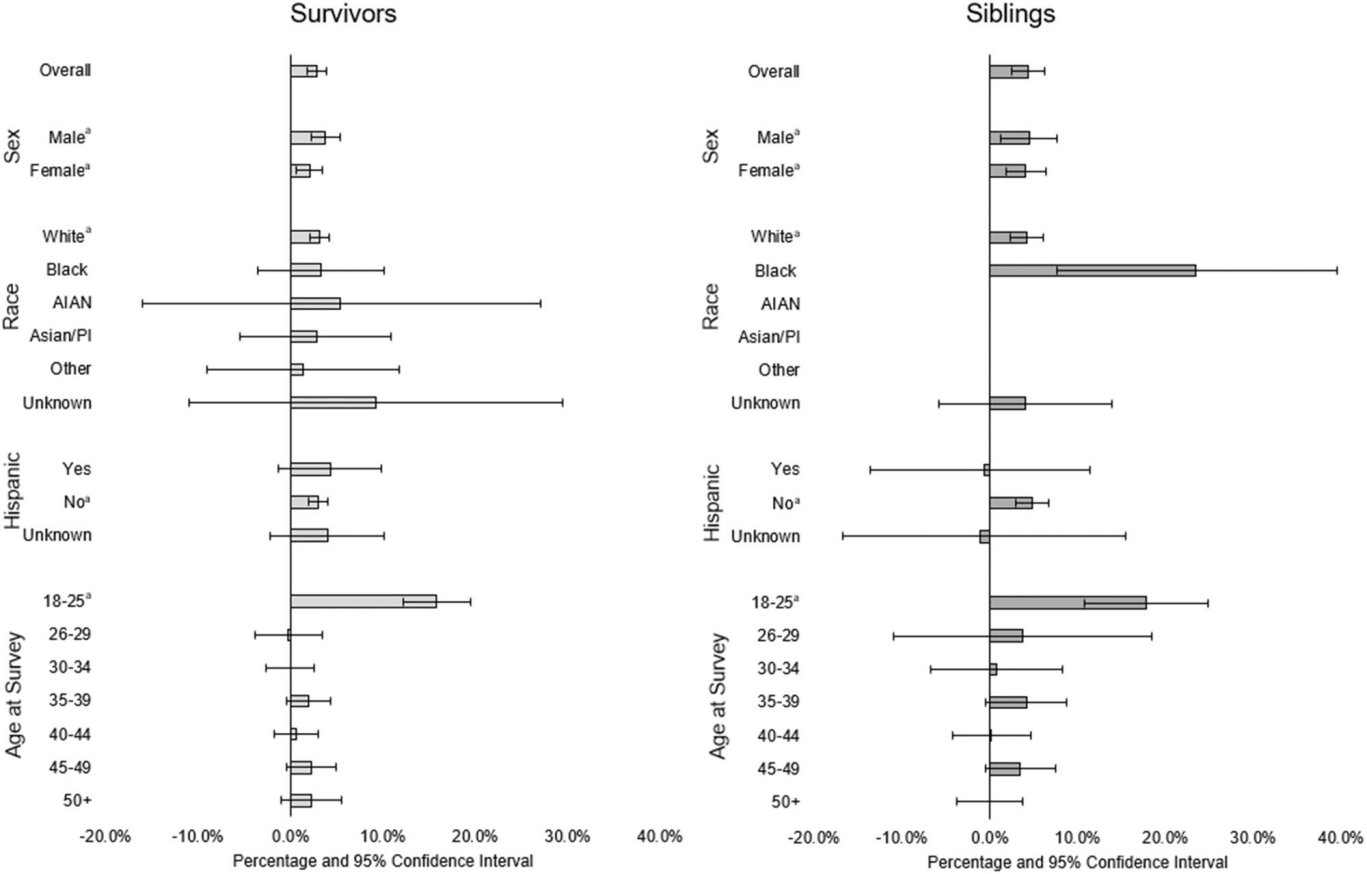
Changes in percent insured overall and by demographic factors among childhood cancer survivors and siblings pre- (2007-2009) to post- (2017-2019) Affordable Care Act (ACA) implementation. Higher percentages indicate an increase in the proportion insured between pre-ACA and post-ACA. Estimates are unadjusted. There were insufficient numbers of siblings (≤20) in the American Indian and Alaska Native, Asian and Pacific Islander, and Other race groups to examine change in insurance coverage. Other race and ethnicity indicates participants indicating more than one race. ^a^*P*** **value less than .05 comparing pre- with post-ACA changes in percent insured. AIAN = Alaska Native; PI = Pacific Islander.

The proportion of insured survivors was statistically significantly higher post-ACA (+2.9%; 89.1% pre-ACA; 92.0% post-ACA; *P* < .05; Figure 1, Table 2), with higher levels of coverage post-ACA among the original cohort (original = 93.7% vs expansion = 90.5%; not shown). When limited to survivors completing both surveys (Supplementary Table 2, available online), the overall increase in coverage was similar (+2.7%; 91.6% pre-ACA; 94.2% post-ACA; *P* < .05). The prevalence of siblings with insurance coverage was statistically significantly higher by 4.4% (90.9% pre-ACA; 95.3% post-ACA; *P* < .05; Figure 1, Table 2). Siblings who completed both surveys did not statistically differ pre- and post-ACA (+1.9%; 93.0% pre-ACA; 94.9% post-ACA; Supplementary Table 2, available online). The proportion insured among most subgroups of survivors and siblings was higher at post-ACA vs pre-ACA (Table 2), with improvements largely among survivors (+15.8%) and siblings (+17.8%) aged 18-25 years (vs <2.3% and <4.2%, respectively for older participants), siblings racialized as Black (+23.5%), and survivors with incomes less than $20 000 (+10.6%) and siblings with incomes less than $20 000 (+14.0%) and $20 000-$39 999 (+10.8%).

**Table 2.** Insurance coverage and type of insurance pre- (2007-2009) vs post- (2017-2019) Affordable Care Act (ACA) implementation among survivors and siblings by demographic subgroups

Demographics	Survivors							Siblings						
	Pre-ACA		Post-ACA				Overall % change in insured	Pre-ACA		Post-ACA				Overall % change in insured
	(n = 7505)^a^		(n = 4030)^b^					(n = 2175)^a^		(n = 987)^b^				
	No.	% insured	No.	% insured	Among insured			No.	% insured	No.	% insured	Among insured		
					% private^c^	% public^c^						% private^c^	% public^c^	
**Overall**	**6687**	**89.1**	**3724**	**92.0**	**81.6**	**18.4**	**+2.9** ^d^	**1978**	**90.9**	**941**	**95.3**	**93.1**	**6.9**	**+4.4** ^d^
Sex														
Male	3286	88.0	1791	91.7	82.8	17.2	+3.7^d^	900	89.3	392	93.8	94.3	5.7	+4.5^d^
Female	3401	90.2	1933	92.3	80.6	19.4	+2.1^d^	1078	92.4	549	96.5	92.2	7.8	+4.1^d^
Race and ethnicity^e^														
Black, non-Hispanic	182	80.2	162	83.9	62.9	37.1	+3.7	39	76.5	15	100.0	86.7	13.3	+23.5
Hispanic	265	82.0	231	86.3	78.7	21.3	+4.3	54	88.5	29	87.9	93.1	6.9	−0.6
Other	406	88.1	181	90.3	79.5	20.5	+2.2	102	87.2	54	88.5	94.4	5.6	+1.3
White, non-Hispanic	5834	89.8	3150	93.0	82.9	17.1	+3.2^d^	1783	91.6	843	96.0	93.1	6.9	+4.4^d^
Age at survey, y														
18-25	461	80.6	297	96.4	77.1	22.9	+15.8^d^	162	82.2	33	100.0	96.3	3.7	+17.8^d^
26-29	1022	85.2	315	85.0	78.5	21.5	−0.2	224	85.2	24	88.9	100.0	0.0	+3.7
30-34	1371	89.5	557	89.5	82.0	18.0	+0.0	320	89.6	84	90.3	88.1	11.9	+0.7
35-39	1529	89.6	761	91.5	80.0	20.0	+1.9	406	91.6	137	95.8	93.4	6.6	+4.2
40-44	1251	92.5	647	93.1	84.1	15.9	+0.6	373	94.4	159	94.6	94.9	5.1	+0.2
45-49	749	92.0	529	94.3	83.1	16.9	+2.3	288	94.1	163	97.6	93.8	6.2	+3.5
50 and older	304	92.7	618	94.9	84.6	15.4	+2.2	205	95.8	341	95.8	92.2	7.8	+0.0
Household income^f^														
<$20 000	545	69.6	286	80.2	29.5	70.5	+10.6^d^	74	67.3	26	81.3	39.1	60.9	+14.0
$20 000-$39 999	1047	83.3	385	84.5	67.8	32.2	+1.2	201	75.8	58	86.6	70.7	29.3	+10.8^d^
$40 000-$59 999	1119	90.1	428	89.7	87.3	12.7	−0.4	302	89.6	94	94.9	89.0	11.0	+5.3
$60 000-$79 999	1055	94.7	434	96.2	94.2	5.8	+1.5	320	93.8	88	94.6	94.3	5.7	+0.8
$80 000-$99 999	755	95.9	395	93.9	95.4	4.6	−2.0	271	96.8	119	96.7	97.4	2.6	−0.1
≥$100 000	1556	97.9	1163	98.8	97.7	2.3	+0.9	692	98.3	455	97.8	98.9	1.1	−0.5
Missing	592	83.0	633	90.1	64.6	35.4	+7.1^d^	116	85.3	101	93.5	89.8	10.2	+8.2^d^

a Limited to participants who were administered and responded to the 2007-2009 survey (survivors: n = 7505; siblings: n = 2175). Estimates are unadjusted. Bold is to indicate the overall results from the analyses by demographic factors. + are to indicate results where insurance increased (− to indicate decreases).

b Limited to participants who were administered and responded to 2017-2019 survey (survivors: n = 4030; siblings: n = 987).

c Type of insurance was only asked at 2017-2019.

d *P* value less than .05 in unadjusted tests of statistical significance from pre-ACA to post-ACA.

e “Other” designates participants indicating other race groups or multiple race groups.

f For pre-ACA, income from 2007-2009 survey was used, and for post-ACA, income from 2017-2019 survey was used.

In multivariable models for the cross-sectional sample, insurance coverage post- vs pre-ACA was higher for both (survivors: OR = 1.4, 95% confidence interval [CI] = 1.2 to 1.6; siblings: OR = 1.6, 95% CI = 1.2 to 2.3; interaction survivor-sibling term not statistically significant, *P* = .35; Supplementary Table 3, available online). Among the longitudinal sample, estimates included survivors (OR = 1.5, 95% CI = 1.2 to 1.9; siblings: OR = 1.4, 95% CI = 0.9 to 2.1; *P*_interaction_ = .73).

### Post-ACA survivor vs sibling type of insurance coverage, underinsurance, and perceived insurance stability

**Figure 2.**
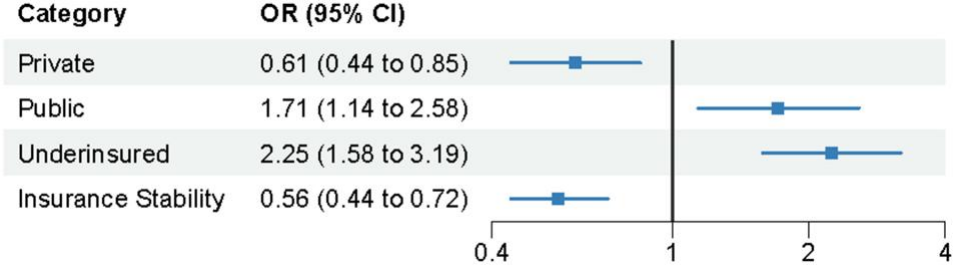
Adjusted odds ratios, 95% confidence intervals, and prevalence for type of insurance (private and public vs uninsured), underinsurance, and perceived insurance stability comparing survivors with siblings post–Affordable Care Act (2017-2019). Limited to participants who were administered and responded to the 2017-2019 survey. All models adjusted for age at survey, sex, and race and ethnicity. Prevalence of insurance estimates includes all participants. Underinsurance and perceived insurance stability include only participants with insurance. Underinsurance calculated among those who reported spending more than 10% of income on medical costs and where family income was reported. Perceived insurance stability indicates participants reporting not concerned or a little concerned about maintaining their insurance coverage. CI = confidence interval; OR = odds ratio.

In the post-ACA period, fewer survivors compared with siblings had private insurance (81.6% [95% CI = 80.5% to 82.8%] vs 93.1% [95% CI = 91.5% to 94.7%]; Table 2). Survivors had 0.61 (95% CI = 0.44 to 0.85; Figure 2) the odds of private insurance vs being uninsured compared with siblings. A higher proportion of survivors had public insurance coverage compared with siblings (18.4% [95% CI = 17.2% to 19.5%] vs 6.9% [95% CI = 5.3% to 8.6%]) Survivors had 1.71 (95% CI = 1.14 to 2.58) the odds of public insurance vs being uninsured in comparison with siblings. Post-ACA, more survivors residing in Medicaid expansion states reported public insurance (17.7%) than survivors in nonexpansion states (13.7%; overall *P* < .001; Table 3). No statistically significant difference was observed among siblings in expansion (6.9%) compared with nonexpansion states (4.8%; overall *P* = .45; Table 3).

**Table 3.** Unadjusted estimates of insurance coverage 2017-2019 among survivors and siblings by residence in medical expansion state or nonexpansion state

Demographics	Medicaid expansion state ^a^			Non-Medicaid expansion state^a^		
	Uninsured	Private	Public	Uninsured	Private	Public
	No. (%)	No. (%)	No. (%)	No. (%)	No. (%)	No. (%)
**Survivors**						
**Total**	**219 (7.4)**	**2283 (74.9)**	**563 (17.7)**	**77 (10.7)**	**620 (75.6)**	**114 (13.7)**
Sex						
Male	122 (8.1)	1117 (75.3)	252 (16.7)	36 (10.6)	301 (77.0)	47 (12.4)
Female	97 (6.7)	1166 (74.5)	311 (18.8)	41 (10.8)	319 (74.3)	67 (14.9)
Race and ethnicity						
Black, non-Hispanic	17 (14.7)	67 (49.6)	46 (35.7)	9 (25.0)	29 (59.5)	9 (15.5)
Hispanic	29 (13.1)	156 (68.8)	46 (18.1)	7 (20.8)	18 (60.1)	4 (19.1)
Other^b^	14 (10.4)	110 (69.7)	31 (19.9)	2 (5.6)	27 (83.2)	4 (11.2)
White, non-Hispanic	159 (6.3)	1950 (77.1)	440 (16.6)	59 (9.5)	546 (77.1)	97 (13.5)
Age at 2017-2019, y						
18-25	11 (4.9)	155 (71.6)	51 (23.5)	1 (1.3)	40 (85.7)	7 (12.9)
26-29	40 (14.7)	186 (67.2)	64 (18.0)	8 (14.4)	43 (66.0)	13 (19.6)
30-34	42 (8.8)	337 (73.3)	97 (17.9)	20 (16.8)	96 (73.1)	16 (10.2)
35-39	51 (7.6)	457 (73.7)	120 (18.7)	17 (10.8)	139 (73.0)	28 (16.2)
40-44	28 (5.3)	420 (79.3)	82 (15.4)	16 (12.9)	105 (74.7)	18 (12.4)
45-49	22 (5.2)	328 (77.0)	76 (17.9)	8 (7.1)	92 (82.2)	12 (10.7)
50 and older	25 (5.0)	400 (80.3)	73 (14.6)	7 (5.3)	105 (79.5)	20 (15.2)
**Siblings**						
**Total**	**34** (**4.5)**	**670** (**88.6)**	**52** (**6.9)**	**11** (**5.9)**	**166** (**89.3)**	**9** (**4.8)**
Sex						
Male	18 (5.7)	279 (88.9)	17 (5.4)	8 (9.0)	77 (86.5)	4 (4.5)
Female	16 (3.6)	391 (88.5)	35 (7.9)	3 (3.1)	89 (91.8)	5 (5.2)
Race and ethnicity						
Black, non-Hispanic	0 (0.0)	6 (75.0)	2 (25.0)	0 (0.0)	7 (100.0)	0 (0.0)
Hispanic	4 (13.8)	23 (79.3)	2 (6.9)	0 (0.0)	3 (100.0)	0 (0.0)
Other^b^	5 (10.2)	41 (83.7)	3 (6.1)	2 (22.2)	7 (77.8)	0 (0.0)
White, non-Hispanic	25 (3.7)	600 (89.6)	45 (6.7)	9 (5.4)	149 (89.2)	9 (5.4)
Age at 2017-2019, y						
18-25	0 (0.0)	23 (95.8)	1 (4.2)	0 (0.0)	3 (100.0)	0 (0.0)
26-29	2 (9.5)	19 (90.5)	0 (0.0)	1 (20.0)	4 (80.0)	0 (0.0)
30-34	8 (11.6)	52 (75.4)	9 (13.0)	1 (4.3)	21 (91.3)	1 (4.3)
35-39	4 (3.5)	101 (89.4)	8 (7.1)	2 (8.0)	23 (92.0)	0 (0.0)
40-44	6 (4.5)	122 (91.7)	5 (3.8)	3 (10.7)	24 (85.7)	1 (3.6)
45-49	3 (2.4)	111 (90.2)	9 (7.3)	1 (2.9)	33 (94.3)	1 (2.9)
50 and older	11 (4.0)	242 (88.6)	20 (7.3)	3 (4.5)	58 (86.6)	6 (9.0)

a Restricted to those without missing information on state of residence and insurance status and type. Bold values indicate the total groups.

b Other designates participants indicating other race groups or multiple race groups.

Among the insured participants, post-ACA, more survivors than siblings reported underinsurance (survivors [9.7%, 95% CI = 8.7% to 10.7%] vs siblings [5.1%, 95% CI = 3.6% to 6.7%]; OR = 2.25, 95% CI = 1.58 to 3.19) (Figure 2). Survivors were less likely to perceive insurance coverage as stable than siblings (survivors [86.3%, 95% CI = 85.2% to 87.3%] vs siblings [91.0%, 95% CI = 89.1% to 92.8%]; OR = 0.56, 95% CI = 0.44 to 0.72).

### Factors associated with post-ACA type of insurance coverage among survivors

**Figure 3.**
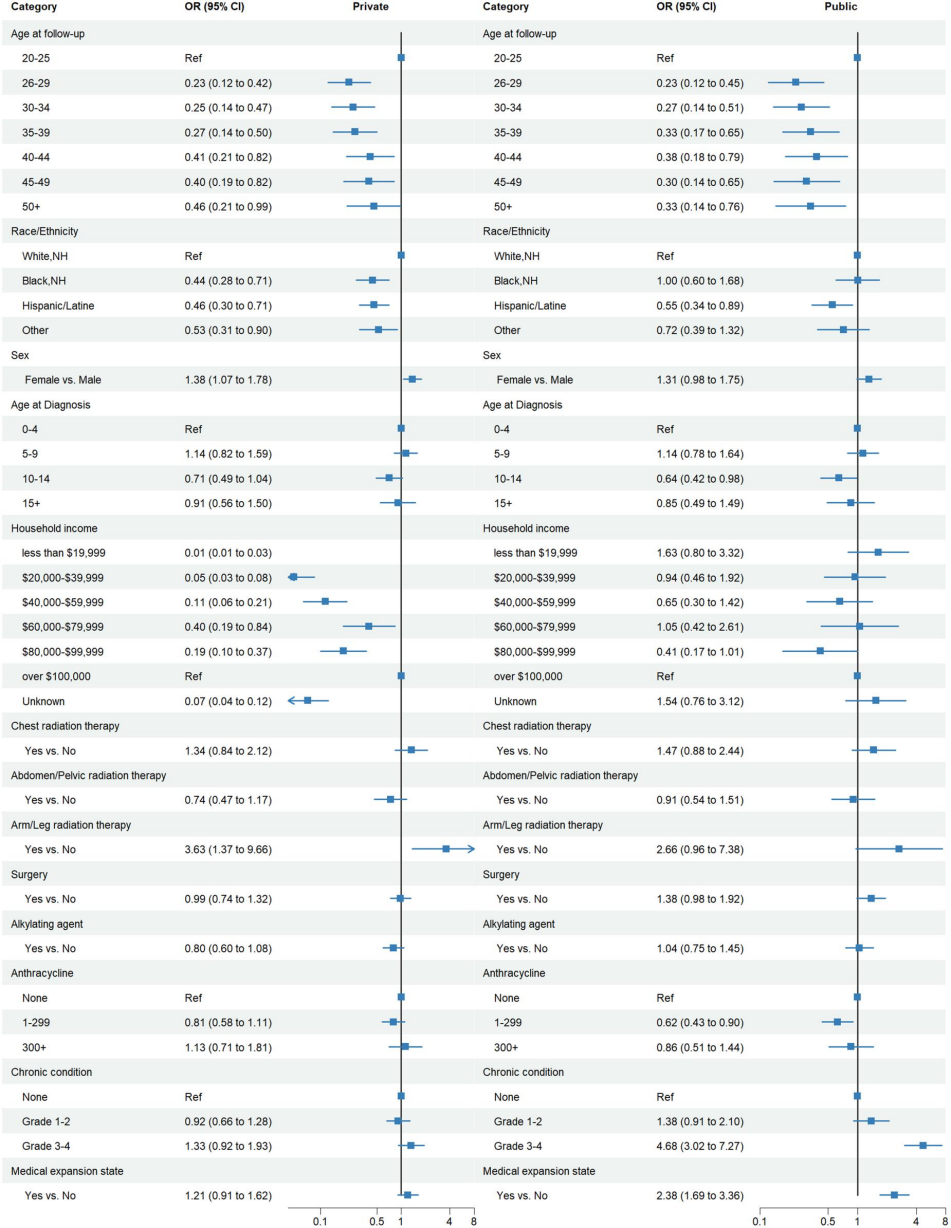
Adjusted odds ratios and 95% confidence intervals for type of insurance coverage (private and public vs uninsured) in association with demographic and clinical factors among childhood cancer survivors post–Affordable Care Act (2017-2019). Multivariable multinomial regression with uninsured as reference. Models limited to participants who were administered and responded to the 2017-2019 survey: survivors (n** **=** **4030). Model covariates include variables statistically significant in univariate analyses at a *P*** **value less than .2. Other race and ethnicity indicate participants indicating more than 1 race. Odds ratios and 95% confidence intervals above 1 indicate statistically significantly higher odds or private or public insurance, respectively, whereas 95% confidence intervals below 1 indicate statistically significantly lower odds of private or public insurance, respectively. CI = confidence interval; NH = non-Hispanic; OR = odds ratio; Ref = referent; RT = radiation therapy.

Compared with survivors aged 18-25 years, those aged 26 years and older were less likely to have public or private insurance post-ACA (Figure 3). Private insurance was less common for all race and ethnic minority groups of survivors compared with non-Hispanic White survivors. Additionally, Hispanic survivors were less likely to have public coverage (OR = 0.6, 95% CI = 0.3 to 0.9) compared with non-Hispanic White survivors. Female survivors were more likely to have private insurance (OR = 1.4, 95% CI = 1.1 to 1.8) than male survivors. Compared with household incomes of $100 000 or more per year, incomes less than $100 000 were less likely to have private insurance. For chronic health conditions, higher grade was associated with increased odds of public insurance (grade 3-4: OR = 4.7, 95% CI = 3.0 to 7.3 vs none) than survivors with no chronic health conditions. Survivors living in Medicaid expansion states were more likely to report public insurance than survivors in states that did not expand Medicaid (OR = 2.4, 95% CI = 1.7 to 3.4).

Among insured survivors (Table 4), the outcomes of underinsured and perceived insurance stability included few statistically significant findings by age subgroups, although the oldest survivors (aged 50 years and older) were less likely to indicate perceived insurance stability in comparison with the youngest survivor group (OR = 0.4, 95% CI = 0.2 to 0.8 vs referent group, survivors 18-25 years). Hispanic survivors were less likely to report their insurance coverage as stable than non-Hispanic White survivors (OR = 0.6, 95% CI = 0.4 to 0.9 vs White survivors). Female survivors were more likely to be underinsured than male survivors (OR = 1.5, 95% CI = 1.1 to 2.1). Underinsurance was more common among those with incomes less than $100 000 per year when compared with the highest household income category (≥$100 000). Middle- to low-income groups were less likely to report their insurance as stable in comparison to the highest income group. Survivors with any chronic health conditions were less likely to perceive their coverage as stable compared with survivors with no chronic health conditions.

**Table 4.** Adjusted odds ratios (ORs) and 95% confidence intervals (CIs) post–Affordable Care Act (2017-2019) of underinsurance and perceived insurance stability among insured childhood cancer survivors

Demographic and clinical characteristics	Underinsurance ^a^^,^^b^		Perceived insurance stability^a^	
	OR (95% CI)	*P*	OR (95% CI)	*P*
Age at 2017-2019, y				
18-25	Referent		Referent	
26-29	2.1 (0.9 to 5.0)	.10	0.7 (0.4 to 1.3)	.23
30-34	1.1 (0.4 to 2.5)	.89	0.6 (0.4 to 1.1)	.10
35-39	1.0 (0.4 to 2.1)	.90	0.7 (0.4 to 1.1)	.14
40-44	0.9 (0.4 to 2.1)	.84	0.6 (0.3 to 1.0)	.05
45-49	1.1 (0.5 to 2.5)	.85	0.6 (0.4 to 1.1)	.09
50 and older	1.4 (0.6 to 3.4)	.41	0.4 (0.2 to 0.8)	.003
Race and ethnicity				
Black, non-Hispanic	N/A		0.7 (0.4 to 1.2)	.24
Hispanic			0.6 (0.4 to 0.9)	.02
Other^c^			0.8 (0.5 to 1.3)	.46
White, non-Hispanic			Referent	
Sex				
Male	Referent		Referent	
Female	1.5 (1.1 to 2.1)	.02	0.9 (0.8 to 1.2)	.63
Age at diagnosis, y				
0-4	Referent		Referent	
5-9	1.3 (0.8 to 2.1)	.24	1.0 (0.8 to 1.4)	.89
10-14	1.5 (0.9 to 2.3)	.11	0.8 (0.6 to 1.1)	.11
15 and older	1.8 (1.1 to 3.0)	.02	1.0 (0.7 to 1.4)	.85
Household income at 2017-2019				
<$20 000	39.2 (20.6 to 74.8)	<.001	0.2 (0.1 to 0.3)	<.001
$20 000-$39 999	25.4 (13.4 to 47.9)	<.001	0.2 (0.1 to 0.3)	<.001
$40 000-$59 999	19.2 (10.2 to 36.0)	<.001	0.3 (0.2 to 0.5)	<.001
$60 000-$79 999	4.6 (2.2 to 9.9)	<.00	0.5 (0.3 to 0.7)	<.001
$80 000-$99 999	2.8 (1.3 to 6.2)	.01	0.7 (0.5 to 1.2)	.19
≥$100 000	Referent		Referent	
Missing	—		0.26 (0.18 to 0.37)	<.001
Chest radiation or total body irradiation				
No	Referent		Referent	
Yes	1.3 (0.9 to 1.8)	.10	0.9 (0.6 to 1.2)	.30
Abdominal or pelvic radiation or total body irradiation				
No	N/A		Referent	
Yes			0.9 (0.7 to 1.3)	.71
Limb, (arm or leg) or total body irradiation				
No	N/A		Referent	
Yes			0.9 (0.6 to 1.4)	.64
Any surgery				
No	N/A		Referent	
Yes			0.9 (0.6 to 1.2)	.30
Alkylating agents				
No	N/A		Referent	
Yes			0.9 (0.7 to 1.1)	.22
Anthracycline cumulative dose				
None	N/A		N/A	
1-299 mg/m^2^				
≥300 mg/m^2^				
Chronic health conditions at 2017-2019				
None	Referent		Referent	
Grade 1-2	1.0 (0.5 to 1.9)	.96	0.6 (0.4 to 0.9)	.02
Grade 3-4	1.6 (0.8 to 2.9)	.15	0.5 (0.4 to 0.8)	.006
Medicaid expansion state resident				
No	Referent		Referent	
Yes	0.9 (0.6 to 1.3)	.48	1.1 (0.8 to 1.4)	.54

a Limited to participants who were administered and responded to the 2017-2019 survey: survivors N = 4030. Model covariates include variables statistically significant in univariate analyses at *P* < .2; covariates for the multivariable models not meeting this significance threshold indicated as not applicable (N/A). Underinsurance was defined as spending >10% of household income on out-of-pocket medical expenses. Perceived insurance stability was generated from an item asking about participants’ concern regarding maintaining current level of insurance coverage.  Statistical significance at *P* < .05.

b Participants missing household income were excluded as required to calculate underinsurance.

c Other designates participants indicating other race groups or multiple race groups.

### Longitudinal changes in insurance coverage among survivors and siblings

Among the longitudinal sample, changes in coverage pre-ACA to post-ACA revealed that age at survey, age at diagnosis, and household income were the statistically significant factors associated with either losing or gaining coverage after the ACA (Table 5). Older survivors were statistically significantly less likely to have lost insurance (ages 45-49 years and 50 years and older: OR = 0.3-0.4 vs ages 30-34 years). Compared with households with incomes of $100 000 or more, survivors and siblings with incomes less than $40 000 per year gained insurance. At the same time, survivors and siblings making up to $60 000 per year lost insurance from pre- to post-ACA in comparison with incomes of $100 000 per year or more. No treatment variables were statistically significant in multivariable models.

**Table 5.** Adjusted odds ratios (ORs) and 95% confidence intervals (CIs) of losing or gaining insurance from pre–Affordable Care Act (ACA; 2007-2009) to post-ACA (2017-2019) implementation among survivors and siblings who completed both pre- and post-ACA surveys^a^

Demographics	Survivors				Siblings				Gained coverage ^a^				Lost coverage^a^			
									Survivors		Siblings		Survivors		Siblings	
	Total	No change	Gained	Lost	Total	No change	Gained	Lost	OR (95% CI)	*P*	OR (95% CI)	*P*	OR (95% CI)	*P*	OR (95% CI)	*P*
Age at 2017-2019, y																
30-34	82	69	2	11	39	29	6	4	Referent		Referent		Referent		Referent	
35-39	317	286	13	18	79	70	1	8	1.4 (0.3 to 6.6)	.67	0.0 (0.0 to 0.4)	.008	0.4 (0.2 to 0.8)	.02	1.2 (0.3 to 4.9)	.80
40-44	353	314	13	26	99	90	5	4	1.5 (0.3 to 6.9)	.64	0.4 (0.1 to 1.7)	.21	0.4 (0.2 to 1.0)	.06	0.7 (0.1 to 3.3)	.65
45-49	356	324	13	19	127	117	3	7	1.6 (0.3 to 8.4)	.57	0.2 (0.0 to 1.1)	.06	0.4 (0.1 to 0.9)	.04	1.3 (0.3 to 5.4)	.75
50 and older	533	493	16	24	299	284	6	9	1.2 (0.2 to 6.8)	.86	0.2 (0.0 to 0.7)	.015	0.3 (0.1 to 0.9)	.04	0.7 (0.2 to 2.8)	.64
Race and ethnicity																
Black, non-Hispanic	30	27	2	1	8	7	0	1	2.7 (0.6 to 12.4)	.19	0.0 (0.0 to 12.8)	.50	0.7 (0.1 to 5.7)	.77	1.9 (0.2 to 20.7)	.59
Hispanic	62	54	2	6	18	17	1	0	1.0 (0.2 to 4.3)	.99	2.8 (0.2 to 33.6)	.41	1.3 (0.5 to 3.3)	.54	0.0 (0.0 to 2.2)	.17
Other^b^	93	84	6	3	36	30	3	3	2.1 (0.9 to 5.3)	.10	3.2 (0.7 to 14.9)	.14	0.6 (0.2 to 2.0)	.41	1.4 (0.3 to 5.3)	.67
White, non-Hispanic	1456	1321	47	88	581	536	17	28	Referent		Referent		Referent		Referent	
Age at diagnosis, y																
0-4	670	607	25	38	N/A	N/A	N/A	NA	Referent		N/A		Referent		N/A	
5-9	352	311	11	30					1.2 (0.5 to 2.7)	.65			2.6 (1.4 to 4.7)	.002		
10-14	332	298	11	23					1.5 (0.6 to 4.1)	.42			2.8 (1.3 to 6.0)	.01		
15 and older	287	270	10	7					2.1 (0.7 to 6.7)	.21			1.0 (0.3 to 3.0)	.99		
Household income^c^																
<$20 000	126	102	5	19	21	16	1	4	3.9 (1.1 to 13.5)	.03	11.4 (0.6 to 225.4)	.11	11.2 (4.3 to 29.5)	<.001	69.9 (6.7 to 732.4)	<.001
$20 000-$39 999	273	220	20	33	63	45	8	10	6.7 (2.5 to 17.7)	<.001	45.1 (5.2 to 392.0)	<.001	9.9 (4.0 to 24.7)	<.001	47.9 (5.9 to 390.4)	<.001
$40 000-$59 999	283	258	9	16	96	81	8	7	2.3 (0.8 to 6.6)	.13	29.1 (3.4 to 250.4)	.002	3.7 (1.4 to 9.6)	.008	17.8 (2.1 to 150.8)	.008
$60 000-$79 999	257	246	5	6	102	95	2	5	1.3 (0.4 to 4.4)	.67	6.3 (0.5 to 74.1)	.14	1.4 (0.4 to 4.5)	.56	12.8 (1.4 to 113.4)	.022
$80 000-$99 999	185	175	6	4	92	89	1	2	2.0 (0.6 to 6.4)	.24	3.4 (0.2 to 56.8)	.39	1.3 (0.4 to 4.8)	.66	5.0 (0.4 to 56.7)	.19
≥$100 000	380	368	6	6	240	238	1	1	Referent		Referent		Referent		Referent	
Missing	137	117	6	14	29	26	0	3	3.8 (1.2 to 12.3)	.03	0.0 (0.0 to 3.3)	.14	7.2 (2.7 to 19.5)	<.001	29.1 (2.8 to 302.9)	.005
Chest radiation	395	366	9	20	N/A	N/A	N/A	NA	0.6 (0.3 to 1.2)	.16	N/A		0.8 (0.5 to 1.3)	.36	N/A	
Any surgery	1267	1153	40	74	N/A	N/A	N/A	N/A	1.0 (0.5 to 1.8)	.94	N/A		1.1 (0.7 to 1.9)	.64	N/A	
Chronic health conditions^c^																
None	237	214	9	14	192	168	9	15	Referent		Referent		Referent		Referent	
Grade 1-2	725	646	35	44	358	330	11	17	1.3 (0.6 to 2.8)	.52	0.9 (0.3 to 2.4)	.76	1.0 (0.5 to 1.9)	.97	0.7 (0.3 to 1.4)	.29
Grade 3-4	679	626	13	40	93	92	1	0	0.5 (0.2 to 1.2)	.12	0.3 (0.0 to 3.0)	.32	0.9 (0.5 to 1.8)	.75	0.0 (0.0 to 0.3)	<.001
Medicaid expansion state resident																
No	339	310	15	14	126	115	5	6	Referent		Referent		Referent		Referent	
Yes	1302	1176	42	84	517	475	16	26	0.7 (0.4 to 1.3)	.30	0.4 (0.1 to 1.2)	.11	1.4 (0.8 to 2.6)	.25	0.9 (0.3 to 2.5)	.88

a Limited to participants who responded to both 2007-2009 survey and the 2017-2019 survey and without missing data for model variables.  Multivariable multinomial logistic regressions were conducted, with no change in insurance as reference. Model covariates include variables statistically significant in univariate analyses at *P* < .2; covariates for the multivariable models not meeting this significance threshold indicated as not applicable (N/A). Statistical significance at *P* < .05.

b Other designates participants indicating other race groups or multiple race groups.

c Household income and chronic conditions as of 2007-2009 survey.

As a supplementary analysis, we examined if changes in coverage differed by whether income did not change, decreased, or increased within the income categories pre- and post-ACA; decreases in income were associated with gains and losses of coverage (Supplementary Table 4, available online).

## Discussion

The ACA expanded health insurance coverage to more than 20 million people and has provided affordable insurance options to millions more in the United States (24). In this first comprehensive study of insurance post-ACA among long-term adult survivors of childhood cancer, we found that survivors and their siblings had a higher proportion with health insurance coverage post-ACA implementation, which supported our hypothesis that both groups would benefit from the ACA. Survivors compared with siblings had public insurance coverage at a higher proportion post-ACA, with larger odds of having public insurance among those residing in Medicaid expansion states. The proportion with insurance coverage increases was higher among survivors aged 18-25 years, likely reflecting the expanded dependent coverage and Medicaid income eligibility provisions of the ACA (25). Yet, underinsurance is of concern, with insured survivors reporting spending proportionally more of their income on medical care than insured siblings.

Our findings identify important insurance coverage improvements for survivors of childhood cancer approximately 10 years after ACA implementation. Prior to the ACA, young adults aged 18-25 years had highest prevalence of being uninsured. We found substantial insurance gains for this age group in our post-ACA analysis, similar to national trends (26). In longitudinal models, middle- and lower-income survivors (making <$60 000 yearly) were more likely to lose insurance after the ACA, although survivors making less than $40 000 yearly also gained insurance. During 2019, the last post-ACA year in our assessment, subsidy qualifications were limited to 100%-400% of the federal poverty level, which for a qualifying individual at this time was a monthly income limit range of $1426-$5704 (approximately $17 112-$68 448 yearly) (27). These results echo national data demonstrating that middle-income individuals are most affected by the lack of subsidies (28). More recently, subsidy expansion has increased insurance enrollment (29), but these subsidies are set to expire in 2026. Zero-premium plans offered on the 2021-2022 marketplace have had frequent turnover, highlighting that even if subsidies make specific plans affordable at enrollment, they may not remain affordable (30).

In our post-ACA analyses, survivors living in Medicaid expansion states were more than 2 times likely to have public insurance than survivors living in nonexpansion states (31). Multiple studies report that Medicaid expansion helped reduce certain disparities in insurance coverage (32,33). However, despite these higher odds of public coverage among all survivors, we found that Hispanic childhood cancer survivors remained less likely to be insured with either public or private coverage. Also, Black and Other race survivors were less likely to have private coverage than their White counterparts. Although most studies of insurance after the ACA among survivors have found reductions in certain racial and ethnic disparities related to insurance coverage (12,32), as of this writing, 9 states have still not expanded Medicaid eligibility to their residents (34). Although the ACA has led to important gains in insurance coverage, insurance disparities may widen for survivors residing in nonexpansion states.

One ACA hallmark is the protections to individuals with preexisting medical conditions, who previously had limited affordable insurance options outside employment or public coverage. In our post-ACA models, survivors with severe grade 3-4 conditions were more likely to have public coverage than survivors with no chronic conditions, but there were no differences for private coverage. However, survivors compared with siblings were more likely to perceive their insurance coverage as not stable. Worries about stability were also more common among survivors with chronic conditions, who were low to middle income or were Hispanic. Insurance stability is of concern as coverage disruptions are associated with decreases in receipt of preventive services, treatment, and survival following cancer diagnosis (13). Thus, although the ACA has protected individuals with health conditions and offered expanded coverage options, worries related to stability of coverage persist for some survivors (21).

Thus, we found that Medicaid and dependent coverage expansion and coverage for individuals with preexisting conditions may have potentially narrowed disparities in insurance coverage but that challenges in insurance access remain. Almost 10% of survivors in our population remain uninsured or underinsured a decade into the ACA (35). At the same time, multiple prior reports demonstrate that post-ACA health care remains unaffordable for many childhood cancer survivors (36,37). Our findings show that, although childhood cancer survivors have higher levels of insurance coverage post-ACA, efforts to improve insurance access for middle-income survivors and those residing in non-Medicaid expansion states may be inadequate, and coverage quality remains a concern (38).

This study has limitations. Because of the largely cross-sectional study design, we cannot causally attribute changes in insurance coverage to the ACA, nor can we disentangle which ACA provision (eg, Medicaid expansion, dependent coverage) directly affected insurance coverage. Our measure of insurance stability was based on participants’ perceptions and may not capture actual interruptions in coverage; at the same time, capturing underinsurance as out-of-pocket costs proportional to income has limitations. However, both provide important markers of health insurance quality and indicate the need for better patient-driven perspectives on insurance cost and quality.

Siblings were used as the comparison group rather than individuals in the general population. As cancer could have affected the economic trajectories of both survivors and their siblings, our results may understate how survivors fare compared with the general population regarding improvements in insurance post-ACA. Our analyses are limited by the pre-ACA survey that did not collect type of health insurance, thus changes in coverage post-ACA should be interpreted with caution. Although the CCSS has launched multiple strategies to maximize the inclusion of racial and ethnic minorities in the cohort (39), this is a largely White population, which might overestimate insurance coverage among the survivor population as a whole. Finally, for the longitudinal analyses, because of size limitations, the estimates for race and ethnicity and household income should be interpreted with caution. However, this study also has numerous strengths including the large cohort, 2 timepoints to evaluate insurance coverage, geographic diversity, and multiple measures of coverage relevant in the post-ACA time frame.

In this national assessment, we found that although insurance coverage for long-term survivors of childhood cancer and siblings improved approximately 10 years after ACA implementation, certain disparities among survivors remain. Survivors—particularly middle to lower income—gained and lost insurance coverage compared with those with higher incomes and perceived their coverage as unstable, which is of concern as coverage disruptions have been linked to poorer receipt of survivorship care (35). In sum, the results of this CCSS study demonstrate the importance of continued efforts to monitor health insurance coverage post-ACA implementation to ensure that all childhood cancer survivors have accessible and affordable coverage to receive needed medical care and maintain healthy lives.

## Supplementary Material

djae111_Supplementary_Data

## Data Availability

The Childhood Cancer Survivor Study (CCSS) is a US National Cancer Institute–funded resource (U24 CA55727) to promote and facilitate research among long-term survivors of cancer diagnosed during childhood and adolescence. CCSS data are publicly available on dbGaP at https://www.ncbi.nlm.nih.gov/gap/ through its accession number phs001327.v2.p1. and on the St Jude Survivorship Portal within the St Jude Cloud at https://survivorship.stjude.cloud/. In addition, utilization of the CCSS data that leverages the expertise of CCSS Statistical and Survivorship research and resources will be considered on a case-by-case basis. For this utilization, a research application of intent followed by an analysis concept proposal must be submitted for evaluation by the CCSS Publications Committee. Users interested in utilizing this resource are encouraged to visit http://ccss.stjude.org. Full analytical data sets associated with CCSS publications since January of 2023 are also available on the St Jude Survivorship Portal at https://viz.stjude.cloud/community/cancer-survivorship-community∼4/publications.
