# Supplementary material for: Health insurance among survivors of childhood cancer following Affordable Care Act implementation
Source: J Natl Cancer Inst. 2024 May 13;116(9):1466–78. doi: 10.1093/jnci/djae111 (PMC11378313; doi:10.1093/jnci/djae111)
Supplement: djae111_Supplementary_Data [file djae111_supplementary_data.pdf]

Supplementary Material

Supplementary Table 1. Demographic and Clinical Factors for Survey Respondents/Non-Respondents

	Survivors				Siblings			
	Pre-ACA Survey		Post-ACA Survey		Pre-ACA Survey		Post-ACA Survey	
	Survey not returned N=1824	Survey returned N=7505	Survey not returned N=1376	Survey returned N=4030	Survey not returned N=115	Survey returned N=2175	Survey not returned N=562	Survey returned N=987
	N (%)	N (%)	N (%)	N (%)	N (%)	N (%)	N (%)	N (%)
**Sex**								
Male	1016 (55.7)	3736 (49.8)	818 (60.5)	1952 (49.4)	105 (48.8)	1008 (46.3)	282 (50.2)	418 (42.4)
Female	808 (44.3)	3769 (50.2)	558 (39.5)	2078 (50.6)	110 (51.2)	1167 (53.7)	280 (49.8)	569 (57.6)
**Race/ethnicity**								
Black, Non-Hispanic	130 (7.1)	227 (3.0)	62 (8.8)	123 (6.6)	9 (4.2)	51 (2.3)	23 (4.1)	15 (1.5)
Hispanic	108 (5.9)	323 (4.3)	75 (9.9)	165 (8.9)	10 (4.7)	61 (2.8)	27 (4.8)	33 (3.3)
Other	137 (7.5)	461 (6.1)	32 (4.6)	69 (4.1)	14 (6.5)	117 (5.4)	30 (5.3)	61 (6.2)
White, Non-Hispanic	1449 (79.4)	6494 (86.5)	1207 (76.7)	3673 (80.5)	182 (84.7)	1946 (89.5)	482 (85.8)	878 (89.0)
**Age at Diagnosis**								
0-4	796 (43.6)	2993 (39.9)	640 (47.8)	1562 (42.3)	--	--	--	--
5-9	421 (23.1)	1630 (21.7)	300 (24.8)	904 (24.6)				
10-14	363 (19.9)	1528 (20.4)	245 (16.2)	907 (20.4)				
15+	244 (13.4)	1354 (18.0)	191 (11.2)	657 (12.7)				
**Diagnosis**								
Leukemia	604 (33.1)	2592 (34.5)	393 (41.1)	1237 (44.1)	--	--	--	--
Central Nervous System	222 (12.2)	881 (11.7)	245 (17.4)	672 (17.9)				
Hodgkin Disease	199 (10.9)	997 (13.3)	128 (7.0)	480 (9.5)				
Non-Hodgkin Lymphoma	157 (8.6)	584 (7.8)	131 (7.9)	349 (6.8)				
Kidney (Wilms)	190 (10.4)	684 (9.1)	145 (7.9)	386 (6.6)				
Neuroblastoma	138 (7.6)	493 (6.6)	140 (9.1)	292 (5.6)				
Soft tissue sarcoma	164 (9.0)	658 (8.8)	96 (4.1)	267 (3.2)				
Bone cancer	150 (8.2)	616 (8.2)	98 (5.4)	347 (6.4)				
**Any Radiation**								
Yes	1006 (64.7)	4547 (65.2)	636 (33.7)	2086 (39.9)	--	--	--	--
No	549 (35.3)	2432 (34.8)	633 (66.3)	1724 (60.1)				
**Any Chemotherapy**								
Yes	1224 (79.2)	5517 (79.7)	1010 (84.5)	3157 (88.1)	--	--	--	--
No	322 (20.8)	1406 (20.3)	254 (15.5)	645 (11.9)				
**Any Surgery**								
Yes	1222 (79.1)	5348 (77.4)	1017 (68.4)	2982 (64.8)	--	--	--	--
No	322 (20.9)	1563 (22.6)	245 (31.6)	818 (35.2)				

Other designates participants indicating other race groups or multiple race groups.

Supplementary Table 2: Insurance Coverage and Type of Insurance Pre-(2007-2009) vs. Post-(2017-2019) ACA Implementation among Survivors and Siblings by Demographic Subgroups: Among Participants Completing Both Surveys

	Survivors N=1840							Siblings N=646						
	Pre-ACA		Post-ACA				Overall % change in insured	Pre-ACA		Post-ACA				Overall % change in insured
					Among Insured							Among insured		
	N	% insured	N	% insured	% Private[c]	% Public[c]		N	% insured	N	% insured	% Private[c]	% Public[c]	
**Overall**	**1685**	**91.6**	**1734**	**94.2**	**81.6**	**18.4**	**+2.7[d]**	**601**	**93.0**	**613**	**94.9**	**93.1**	**6.9**	**+1.9**
**Sex**														
Male	789	91.0	821	94.7	85.6	14.4	3.7[d]	254	92.7	252	92.0	96.0	4.0	-0.7
Female	896	92.1	913	93.8	82.0	18.0	1.7	347	93.3	361	97.0	91.5	8.5	3.7[d]
**Race/ethnicity**														
Black, NH	38	86.4	40	90.9	69.2	30.8	4.5	--	--	--	--	--	--	--
Hispanic	61	81.3	67	89.3	80.6	19.4	8.0	16	88.9	15	83.3	93.3	6.7	-5.6
Others	102	97.1	97	92.4	89.5	10.5	-4.7	31	86.1	31	86.1	93.5	6.5	0.0
White, NH	1484	91.8	1530	94.7	83.9	16.1	2.9[d]	547	93.7	559	95.7	93.3	6.7	2.0[d]
**Age at Survey**														
18-25	131	85.1	--	--	--	--	--	43	84.3	--	--	--	--	--
26-29	259	90.9	--	--	--	--	--	55	85.9	--	--	--	--	--
30-34	358	92.0	87	92.6	75.6	24.4	0.6	92	94.8	32	82.1	97.3	2.7	-12.7
35-39	378	91.5	328	92.4	82.2	17.8	0.9	123	93.9	76	96.2	95.6	4.4	2.3
40-44	312	93.1	370	94.1	84.3	15.7	1.0	119	94.4	92	92.9	94.3	5.7	-1.5
45-49	182	92.9	390	94.7	83.2	16.8	1.8	97	95.1	124	96.9	91.8	8.2	1.8
50+	65	95.6	559	95.4	85.9	14.1	-0.2	72	96.0	288	96.0	92	8	0.0
**Household Income[e]**														
<$20,000	114	79.7	114	83.8	20.4	79.6	4.1	16	76.2	15	78.9	21.4	78.6	2.7
$20,000-$39,999	260	85.2	161	85.2	66.3	33.8	0.0	47	74.6	37	88.1	73.0	27.0	13.5
$40,000-$59,999	285	91.6	197	91.6	86.2	13.8	0.0	88	91.7	51	91.1	87.8	12.2	-0.6
$60,000-$79,999	278	95.9	192	97.0	91.7	8.3	1.1	95	92.2	60	93.8	93.2	6.8	1.6
$80,000-$99,999	200	96.2	202	95.3	95.0	5.0	-0.9	90	97.8	73	96.1	98.6	1.4	-1.7
Over $100,000	411	97.4	627	99.4	98.2	1.8	2.0[d]	239	98.8	311	97.2	98.7	1.3	-1.6
Missing	133	84.7	241	93.1	69.5	30.5	8.4[d]	26	89.7	66	95.7	93.8	6.3	6.0

[a]Limited to participants who were administered and responded to the 2007-2009 survey: survivors N=7505; siblings N=2175.

[b]Limited to participants who were administered and responded to 2017-2019 survey: survivors N=4030 siblings N=987.

[c]Type of insurance was only asked at 2017-2019.

[d]P<0.05 in unadjusted tests of significance from pre-ACA to post-ACA

Estimates are unadjusted and limited to participants completing both the Pre-/Post-ACA surveys. There were insufficient numbers of participants (≤20) among Black siblings to estimate changes. Other designates participants indicating other race groups or multiple race groups. For post-ACA, when restricted to survivors and siblings who completed both surveys, all participants were 30 years of age or older.

Supplementary Table 3. Adjusted Odds Ratios and 95% Confidence Intervals of Survivor vs. Sibling Insurance Coverage Comparisons Pre-ACA (2007-2009) to Post-ACA (2017-2019)[a]

	**Survivor**			**Siblings**			**Test of Survivor/Sibling Difference in Changes[b]**
**Cross-sectional**	Pre-ACA Survey N=7505	Post-ACA Survey N=4030	Change in percent	Pre-ACA Survey N=2175	Post-ACA Survey N=987	Change in percent	
**% insured**	89.1	92.0	2.9 (1.8-3.9)	90.9	95.3	4.4 (2.5-6.3)	
	Post- vs. pre-ACA Odds ratio 1.4 (1.2-1.6)			Post- vs. pre- ACA Odds ratio 1.6 (1.2-2.3)			P-value=0.35
**Longitudinal**	Pre-ACA Survey N=1840	Post-ACA Survey N=1840	Change in percent	Pre-ACA Survey N=646	Post-ACA Survey N=646	Change in percent	
**% insured**	91.6	94.2	2.7 (1.0-4.4)	93.0	94.9	1.9 (-0.9-4.6)	
	**Post- vs. pre-ACA Odds Ratio/95% CI** 1.5 (1.2-1.9)			**Post- vs. pre-ACA Odds Ratio/95% CI** 1.4 (0.9-2.1)			P-value=0.73

[a]Models adjusted for age at survey, sex, and race/ethnicity.
[b]Interaction term
Cross-sectional samples include participants from the 2007-2009 survey, survivors diagnosed in 1970-86: N=7,505 and siblings: N=2,175) and (2017-2019 survey, survivors diagnosed in 1970-99: N=4,030 and siblings: N=987). Longitudinal sample includes participants who responded to both 2007-2009 survey and the 2017-2019 survey: survivors N=1840; siblings N=646.

Supplementary Table 4. Unadjusted Estimates of Insurance Coverage 2017-2019 among Survivors and Siblings by Residence in Medical Expansion State or Non-Expansion State

	Medicaid Expansion State						Non-Medicaid Expansion State					
	Uninsured		Private		Public		Uninsured		Private		Public	
**Survivors**	**N**	**%**	**N**	**%**	**N**	**%**	**N**	**%**	**N**	**%**	**N**	**%**
**Total**	**219**	**7.4**	**2283**	**74.9**	**563**	**17.7**	**77**	**10.7**	**620**	**75.6**	**114**	**13.7**
**Sex**												
Male	122	8.1	1117	75.3	252	16.7	36	10.6	301	77.0	47	12.4
Female	97	6.7	1166	74.5	311	18.8	41	10.8	319	74.3	67	14.9
**Race**												
White	177	6.5	2106	77.1	471	16.5	67	10.0	572	77.0	98	13.0
Black	19	15.2	71	49.7	48	35.1	9	24.2	30	59.2	10	16.7
AIAN	3	23.8	7	40.7	8	35.5	0	0.0	3	75.0	1	25.0
Asian/PI	2	6.7	36	75.0	10	18.3	0	0.0	5	88.4	1	11.6
Other	16	17.2	55	60.0	23	22.8	1	6.3	9	58.1	3	35.6
Unknown	2	15.4	8	61.5	3	23.1	0	0.0	1	50.0	1	50.0
**Hispanic**												
Yes	29	13.1	156	68.8	46	18.1	7	20.8	18	60.1	4	19.1
No	186	6.9	2074	75.3	510	17.8	68	10.3	586	76.1	108	13.6
Unknown	4	6.3	53	82.8	7	10.9	2	10.0	16	80.0	2	10.0
**Age at 2017-2019**												
18-25	11	4.9	155	71.6	51	23.5	1	1.3	40	85.7	7	12.9
26-29	40	14.7	186	67.2	64	18.0	8	14.4	43	66.0	13	19.6
30-34	42	8.8	337	73.3	97	17.9	20	16.8	96	73.1	16	10.2
35-39	51	7.6	457	73.7	120	18.7	17	10.8	139	73.0	28	16.2
40-44	28	5.3	420	79.3	82	15.4	16	12.9	105	74.7	18	12.4
45-49	22	5.2	328	77.0	76	17.9	8	7.1	92	82.2	12	10.7
≥50	25	5.0	400	80.3	73	14.6	7	5.3	105	79.5	20	15.2
**Siblings**	**N**	**%**	**N**	**%**	**N**	**%**	**N**	**%**	**N**	**%**	**N**	**%**
**Total**	**34**	**4.5**	**670**	**88.6**	**52**	**6.9**	**11**	**5.9**	**166**	**89.3**	**9**	**4.8**
**Sex**												
Male	18	5.7	279	88.9	17	5.4	8	9.0	77	86.5	4	4.5
Female	16	3.6	391	88.5	35	7.9	3	3.1	89	91.8	5	5.2
**Race**												
White	29	4.2	622	89.2	46	6.6	9	5.3	151	89.3	9	5.3
Black	0	0.0	6	75.0	2	25.0	0	0.0	7	100.0	0	0.0
AIAN	0	0.0	2	100.0	0	0.0	0	0.0	1	100.0	0	0.0
Asian/PI	1	16.7	5	83.3	0	0.0	1	100.0	0	0.0	0	0.0
Other	3	25.0	8	66.7	1	8.3	0	0.0	3	100.0	0	0.0
Unknown	1	3.2	27	87.1	3	9.7	1	20.0	4	80.0	0	0.0
**Hispanic**												
Yes	4	13.8	23	79.3	2	6.9	0	0.0	3	100.0	0	0.0
No	27	3.9	619	89.3	47	6.8	10	5.6	158	89.3	9	5.1
Unknown	3	8.8	28	82.4	3	8.8	1	16.7	5	83.3	0	0.0
**Age at 2017-2019**												
18-25	0	0.0	23	95.8	1	4.2	0	0.0	3	100.0	0	0.0
26-29	2	9.5	19	90.5	0	0.0	1	20.0	4	80.0	0	0.0
30-34	8	11.6	52	75.4	9	13.0	1	4.3	21	91.3	1	4.3
35-39	4	3.5	101	89.4	8	7.1	2	8.0	23	92.0	0	0.0
40-44	6	4.5	122	91.7	5	3.8	3	10.7	24	85.7	1	3.6
45-49	3	2.4	111	90.2	9	7.3	1	2.9	33	94.3	1	2.9
≥50	11	4.0	242	88.6	20	7.3	3	4.5	58	86.6	6	9.0

Restricted to those without missing information on state of residence and insurance status/type
American Indian/Alaska Native (AIAN); Asian/Pacific Islander (PI)

Supplementary Table 5. Adjusted Odds Ratios and 95% Confidence Intervals of Losing or Gaining Insurance from Pre-ACA (2007-2009) to Post ACA (2017-2019) Implementation among Survivors and Siblings who completed both Pre- and Post-ACA Surveys: Changes in Insurance[a]

	Losing Insurance				Gaining Insurance			
	Survivors		Siblings		Survivors		Siblings	
	OR (95% CI)	P	OR (95% CI)	P	OR (95% CI)	P	OR (95% CI)	P
**Age at 2017-2019**								
30-34	Ref		Ref		Ref		Ref	
35-39	2.3 (0.3 - 18.4)	0.44	0.0 (0.0 – 0.4)	<.001[d]	0.3 (0.1 - 0.7)	0.006[d]	0.7 (0.2 - 2.5)	0.54
40-44	1.4 (0.2 - 12.5)	0.74	0.2 (0.1 - 0.9)	0.038[d]	0.3 (0.1 - 0.9)	0.026[d]	0.3 (0.1 - 1.2)	0.08
45-49	1.4 (0.1 - 13.0)	0.78	0.1 (0.0 - 0.6)	0.007[d]	0.2 (0.1 - 0.7)	0.008[d]	0.4 (0.1 - 1.6)	0.20
≥50	1.0 (0.1 - 10.6)	0.99	0.1 (0.0 - 0.3)	<.001[d]	0.2 (0.1 - 0.7)	0.011[d]	0.1 (0.0 - 0.6)	0.006[d]
**Race/Ethnicity**								
Black, Non-Hispanic	1.4 (0.2 - 11.1)	0.77	0.0 (0.0 – 13.7)	0.54	0.8 (0.1 - 6.1)	0.81	3.9 (0.4 - 38.9)	0.21
Hispanic	1.5 (0.3 - 6.9)	0.57	1.8 (0.1 - 22.9)	0.63	0.9 (0.3 - 3.1)	0.89	0.0 (0.0 – 2.7)	0.98
Other[b]	1.6 (0.5 - 4.9)	0.38	3.5 (0.8 - 15.0)	0.10	0.7 (0.2 - 2.4)	0.59	1.5 (0.4 - 5.7)	0.52
White, Non-Hispanic	Ref		Ref		Ref		Ref	
**Age at Diagnosis**								
0-4	Ref		NA		Ref		NA	
5-9	1.0 (0.4 - 2.5)	0.92	NA		1.7 (0.8 - 3.4)	0.14	NA	
10-14	1.0 (0.3 - 3.5)	0.99	NA		1.8 (0.7 - 4.2)	0.20	NA	
15+	2.3 (0.6 - 9.0)	0.23	NA		0.9 (0.3 - 2.8)	0.83	NA	
**Household income change from pre-ACA to post-ACA**								
No change	Ref	Ref			Ref		Ref	
Decrease	2.7 (1.3 - 5.6)	0.009[d]	6.3 (1.5 - 25.4)	0.010[d]	2.3 (1.2 - 4.4)	0.013[d]	2.5 (0.8 - 7.6)	0.10
Increase	0.6 (0.2 - 1.3)	0.16	3.2 (0.9 - 11.3)	0.07	1.3 (0.8 - 2.4)	0.32	1.4 (0.5 - 3.4)	0.52
**Chest Radiation**	0.7 (0.3 - 1.7)	0.45	NA		0.9 (0.5 - 1.7)	0.83	NA	
**Any Surgery**	0.6 (0.3 - 1.3)	0.22	NA		0.8 (0.5 - 1.4)	0.47	NA	
**Chronic Health Conditions[b]**								
None	Ref		Ref		Ref		Ref	
Grade 1-2	1.8 (0.7 - 4.8)	0.25	0.6 (0.2 - 1.5)	0.26	1.0 (0.5 - 2.0)	0.99	0.7 (0.3 - 1.6)	0.43
Grade 3-4	0.7 (0.2 - 2.1)	0.51	0.4 (0.0 - 3.3)	0.37	0.9 (0.4 - 1.9)	0.77	0.0 (0.0 – 0.5)	0.008[d]
**Medicaid Expansion State Resident**								
No	Ref		Ref		Ref		Ref	
Yes	0.8 (0.4 - 1.6)	0.46	0.4 (0.1 - 1.4)	0.15	1.5 (0.8 - 3.0)	0.21	1.3 (0.5 - 3.5)	0.66

[a]Multivariable multinomial logistic regressions were conducted, with no change in insurance as reference.
[b]Chronic conditions as of 2007-2009 survey.
Limited to participants who responded to both 2007-2009 survey and the 2017-2019 survey: survivors N=1840; siblings N=646. Model covariates include variables significant in univariate analyses at p<0.2; covariates for the multivariable models not meeting this significance threshold indicated as not applicable (N/A). Other designates participants indicating other race groups or multiple race groups. Statistical significance at p<0.05.

Supplementary Figure 1: Study Sample and Response Rates

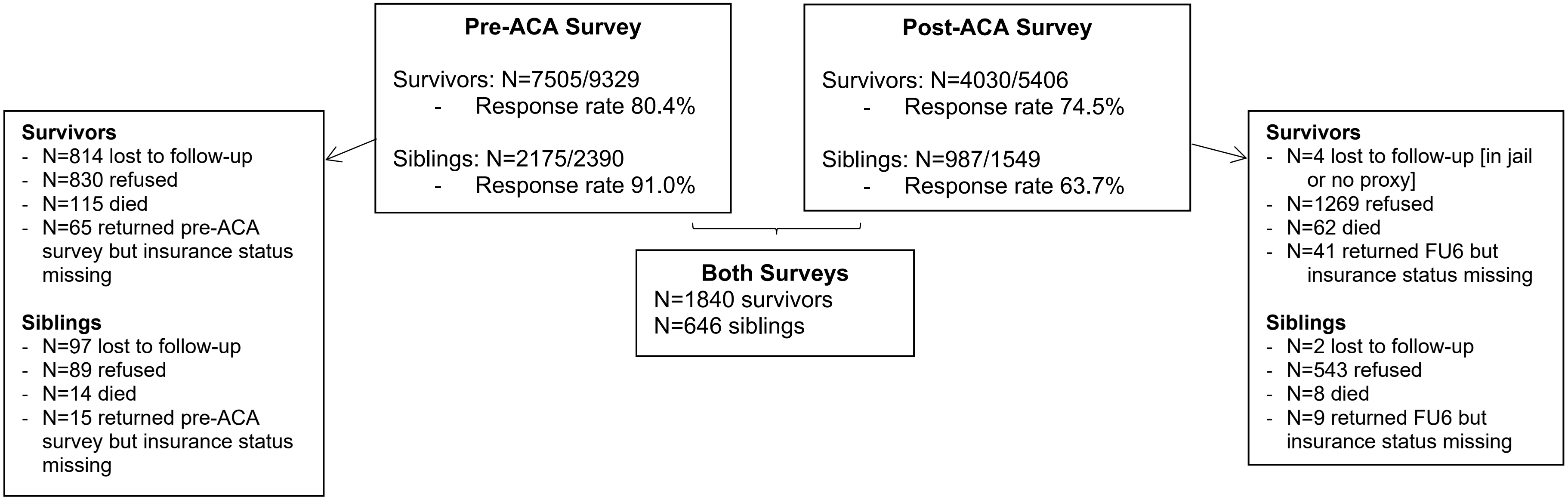

Footnote: The response rates are calculated by excluding Canadian participants and survivors <18 years old. The pre-ACA survey only included the original cohort. The post-ACA questionnaire included both the original cohort and expansion cohort, with 3 versions of questionnaire (long, medium, short) sent to survivors, where only the medium version included items on insurance. The post-ACA medium survey was sent to the selected 5486 survivors. Among the 4151 survivors who returned the medium survey, there were n=80 Canadian residents and n=41 did not provide insurance, resulting in N=4030.
